# Renal primary cilia lengthen in the progression of diabetic kidney disease

**DOI:** 10.3389/fendo.2022.984452

**Published:** 2022-11-17

**Authors:** Yunfeng Bai, Ping Li, Jiaona Liu, Lu Zhang, Shaoyuan Cui, Cuiting Wei, Bo Fu, Xuefeng Sun, Guangyan Cai, Quan Hong, Xiangmei Chen

**Affiliations:** ^1^ Department of Nephrology, First Medical Center of Chinese People's Liberation Army (PLA) General Hospital, Nephrology Institute of the Chinese People’s Liberation Army, State Key Laboratory of Kidney Diseases, National Clinical Research Center for Kidney Diseases, Beijing Key Laboratory of Kidney Disease Research, Beijing, China; ^2^ Department of Nephrology, The First Affiliated Hospital of Xiamen University, School of Medicine, Xiamen University, Xiamen, China; ^3^ Institute of Chinese Medicine, Guangdong Pharmaceutical University, Guangzhou, China

**Keywords:** diabetic kidney disease, primary cilia, renal tubular epithelial cell, ciliotherapy, therapeutic target

## Abstract

Diabetic kidney disease (DKD) is the leading cause of end-stage renal disease, and its early pathogenesis is critical. Shear stress caused by glomerular hyperfiltration contributes to the initiation of kidney injury in diabetes. The primary cilium of renal tubular epithelial cells (RTECs) is an important mechanical force sensor of shear stress and regulates energy metabolism homeostasis in RTECs to ensure energy supply for reabsorption functions, but little is known about the alterations in the renal cilium number and length during the progression of DKD. Here, we demonstrate that aberrant ciliogenesis and dramatic increase in the cilium length, the number of ciliated cells, and the length of cilia are positively correlated with the DKD class in the kidney biopsies of DKD patients by super-resolution imaging and appropriate statical analysis methods. This finding was further confirmed in STZ-induced or db/db diabetic mice. These results suggest that the number and length of renal cilia may be clinically relevant indicators and that cilia will be attractive therapeutic targets for DKD.

## 1 Introduction

Diabetic kidney disease (DKD) is a chronic progressive disorder that can lead to kidney failure and is currently the leading cause of kidney replacement therapy (1). The International Diabetes Federation reports that 537 million adults are living with diabetes, and the number is predicted to increase to 643 million by 2030 and 783 million by 2045 (2). Current therapy for DKD is limited to strict glycemic control and blood pressure control with angiotensin-converting enzyme (ACE) inhibitors or angiotensin-receptor blockers (ARBs) and sodium-glucose cotransporter 2 (SGLT2) inhibitors (3). Glomerular hyperfiltration drives early DKD pathogenesis (4, 5). Revealing how glomerular hyperfiltration accelerates the progression of DKD is important to its early prevention.

The kidney is the second most energy-demanding organ (next to the heart) in the human body (6). The primary cilia in renal tubular epithelial cells (RTECs) play fundamental roles in sensing shear stress induced by biological fluids and then stimulating mitochondrial biogenesis and metabolic reprogramming to ensure energy production during glucose reabsorption (7). In the presence of glomerular ultrafiltrate, the primary cilia of RTECs initiate lipophagy and produce free fatty acids as fuel; the phosphorylation of PGC1α stimulates abundant mitochondrial factories to allow fatty acids to enter mitochondria for β oxidation and generate ATP, thus ensuring sufficient energy supply for reabsorption. Both of the two key signaling pathways are mediated by AMPK.

The primary cilium is an antenna-like, microtubule-based immotile organelle projecting from the surface of most vertebrate cell types that detects and transmits extracellular signals to regulate diverse cellular processes during development and to maintain tissue homeostasis (8, 9). A characteristic marker for primary cilia is the acetylation of axonemal tubulin (10). The ciliary life cycle is closely related to the cell cycle (11). Briefly, cilia assembly occurs in the G0/G1 phase, whereas cilia must be disassembled prior to mitosis. A precise dynamic balance between cilia assembly and disassembly is essential for appropriate embryonic development and homeostasis. Disruption of the balance underlies a pleiotropic group of diseases termed ciliopathies, which affect either a single organ or multiple organs, characterized by the following phenotypes: retinal degradation, hearing loss, malformation of the central nervous system, and polycystic kidney (12, 13). The significance of the renal cilium is reinforced by the defect in cilia that leads to polycystic kidney disease, Meckel–Gruber syndrome (MKS), Bardet–Biedl syndrome (BBS), nephronophthisis (NPHP), and renal cell carcinoma (RCC) (14–16).

The glomeruli of DKD patients are continuously in a hyperfiltration state, and the abnormal lipid metabolism of RTECs is an important pathological basis for the progression of DKD. That is, the lipolysis pathway of RTECs is defective; the decomposition of triglycerides into free fatty acids and the mitochondrial β-oxidation to produce the ATP pathway are blocked, which induces cell apoptosis and dedifferentiation and accelerates the process of renal fibrosis (17–19). However, little was known about the function of primary cilia in DKD, and how glomerular hyperfiltration in the early phase of DKD contributes to the execution of primary cilia is poorly understood.

Here, we demonstrated that the renal cilium length and number were positively correlated with the DKD class. Similar results were further confirmed in streptozotocin (STZ)-induced diabetic mice and db/db mice, suggesting that aberrant cilium elongation may accelerate DKD progression. Thus, our study indicates that proper cilia dynamics are crucial in the early progression from diabetes to DKD.

## 2 Materials and methods

### 2.1 Human renal biopsy samples

Renal biopsies were performed as part of routine clinical diagnostic investigations and were collected as described in **Supplementary Table S1**. Patients with other known kidney diseases were excluded. The renal biopsy samples were obtained from the Department of Nephrology of The First Affiliated Hospital of Xiamen University. Control kidney samples (*n* = 3) were obtained from the healthy kidney poles of individuals without diabetes or kidney diseases who underwent tumor nephrectomies. The investigations were conducted in accordance with the principles of the Declaration of Helsinki and were approved by the Research Ethics Committee of Xiamen University after informed consent was obtained from the subjects.

All renal biopsy samples that were diagnosed as DKD were classified in accordance with a new pathologic classification provided by the Renal Pathology Society (20). From mild to severe, DKD was divided into three hierarchical glomerular lesions as follows: Class I, glomerular basement membrane thickening, isolated glomerular basement membrane thickening, and only mild and nonspecific changes by light microscopy that did not meet the criteria of Classes II through IV; Class II, mild (IIa) or severe (IIb) mesangial expansion, glomeruli classified as mild or severe mesangial expansion but without nodular sclerosis (Kimmelstiel–Wilson lesions) or global glomerulosclerosis in more than 50% of glomeruli; and Class III, nodular sclerosis (Kimmelstiel–Wilson lesions), at least one glomerulus with a nodular increase in the mesangial matrix (Kimmelstiel–Wilson).

### 2.2 Mouse models

#### 2.2.1 STZ-induced diabetic mouse model

Male mice aged 8 weeks were administered with streptozotocin (STZ, Sigma-Aldrich, St. Louis, MO) by intraperitoneal injection after a 6-h fast for five consecutive days (50 mg/kg per day). One week after the last injection, the blood glucose levels were measured. Diabetes was confirmed by a fasting blood glucose level > 300 mg/dl. Age- and sex-matched mice injected with citrate vehicle served as controls. All mice were euthanized at 16 or 24 weeks after STZ or vehicle injection. Body weight and blood glucose levels were monitored biweekly. Urine was collected to measure urinary protein and creatinine levels.

Spontaneous type 2 diabetic db/db mice

db/db mice (BKS.Leprem2Cd479/Gpt Stock No. T002407) and age-matched nondiabetic db/m male mice were purchased from GemPharmatech Co., Ltd. (Jiangsu China). These mice developed DKD, which is defined by the development of albuminuria at 10 weeks of age. All animal studies were performed according to the protocol approved by the Animal Care Committee at the First Medical Center of Chinese PLA General Hospital.

### 2.3 Measurements of the UACR

The urine albumin concentration was measured using a mouse albumin ELISA kit (Bethyl Laboratories, Montgomery, TX). Urine creatinine levels in the same samples were measured using a creatinine colorimetric assay kit (Cayman, MI) according to the manufacturer’s instructions. The level of urine albumin was normalized to urine creatinine (UACR).

### 2.4 Antibodies and reagents

The antibodies and reagents used in this study included rabbit anti-ARL13B (1:500, 17711–1-AP; Proteintech, Chicago, USA).

### 2.5 Immunofluorescence microscopy

For tissue immunofluorescence analysis, kidneys were fixed in 4% paraformaldehyde (PFA) overnight and then embedded in paraffin according to standard procedures. Sections were cut at a thickness of 4 μm. The tissue sections were blocked with 3% BSA and 1% normal goat serum in 0.1% Triton X-100/PBS before being incubated with primary antibodies. The secondary antibody was Alexa Fluor 488–conjugated goat anti-rabbit IgG (Thermo Fisher Scientific, Massachusetts, USA). DNA was stained with Hoechst 33342 (1:1,000, H3570; Thermo Fisher Scientific, Massachusetts, USA).

**Figure 1:**
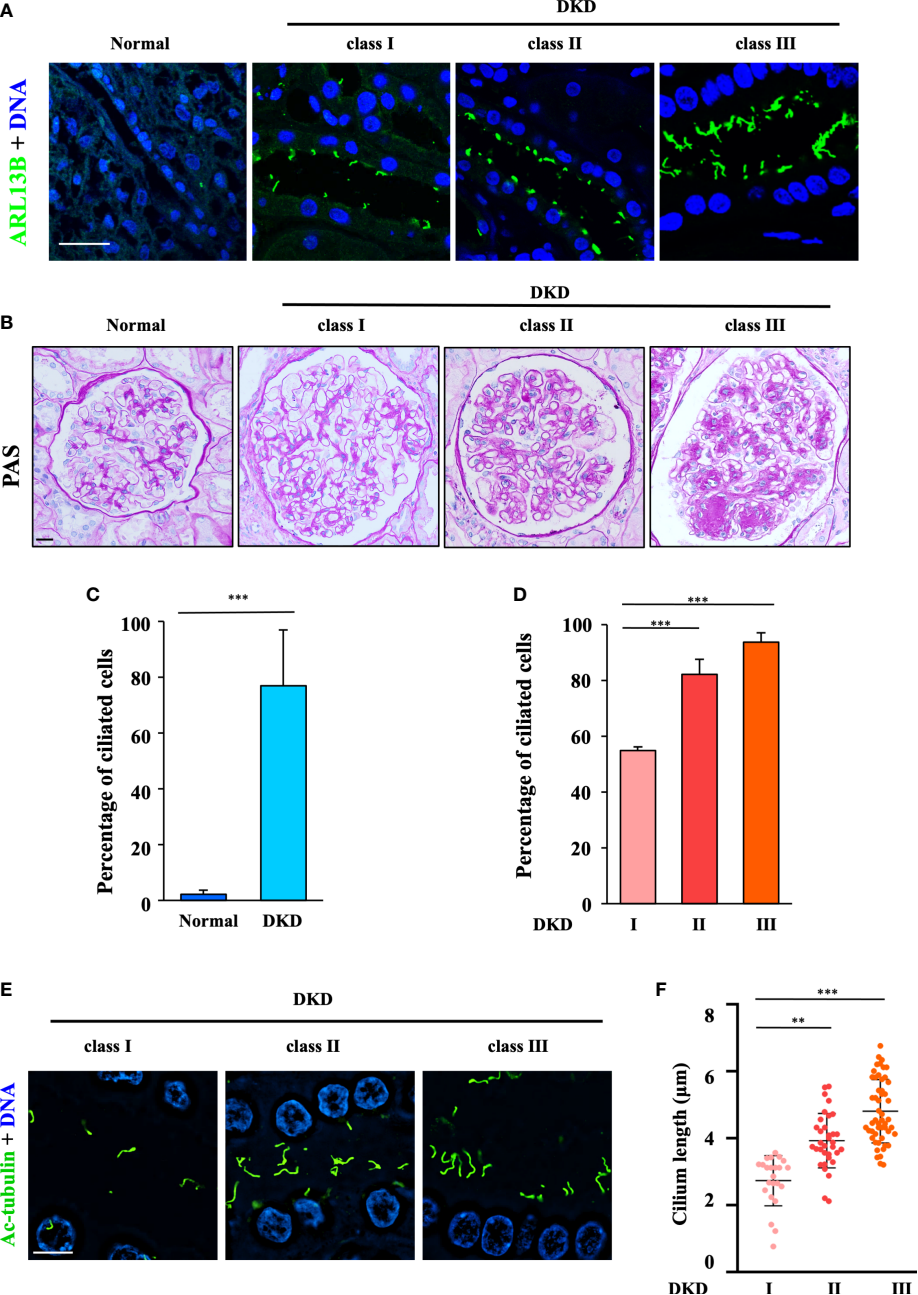
Aberrant ciliogenesis and dramatic increase in the cilium length in humans with diabetic kidney disease. **(A)** Human renal tissue sections were stained for the ciliary marker ARL13B (green) and DNA (blue). Scale bar, 20 μm (three subjects per group). **(B)** Human renal tissue sections from normal kidney poles (*n* = 3); subjects with mild (Class I, *n* = 3), moderate (Class II, *n* = 3), or severe (Class III, *n* = 3) histopathological DKD lesions were stained with periodic acid–Schiff (PAS). Scale bar, 20 μm. **(C)** Quantification of the ciliated cells in all DKD subjects in **(B)**. The data are the means ± SD. An unpaired two-tailed t-test was performed. ***P < 0.001. **(D)** Quantification of ciliated cells in DKD subjects with different classes in **(B)**. The data are the means ± SD. One-way ANOVA was performed followed by Dunnett’s multiple comparisons. **P < 0.01; ***P < 0.001. **(E)** Kidney sections from subjects with different classes of DKD were stained for the ciliary marker ARL13B (green) and DNA (blue) and then imaged using a superresolution microscope. Scale bar, 10 μm. **(F)** Quantification of the cilium length in **(E)**. Each dot represents one cell. The data are the means ± SD. One-way ANOVA was performed followed by Dunnett’s multiple comparisons. **P < 0.01; ***P < 0.001.

**Figure 2:**
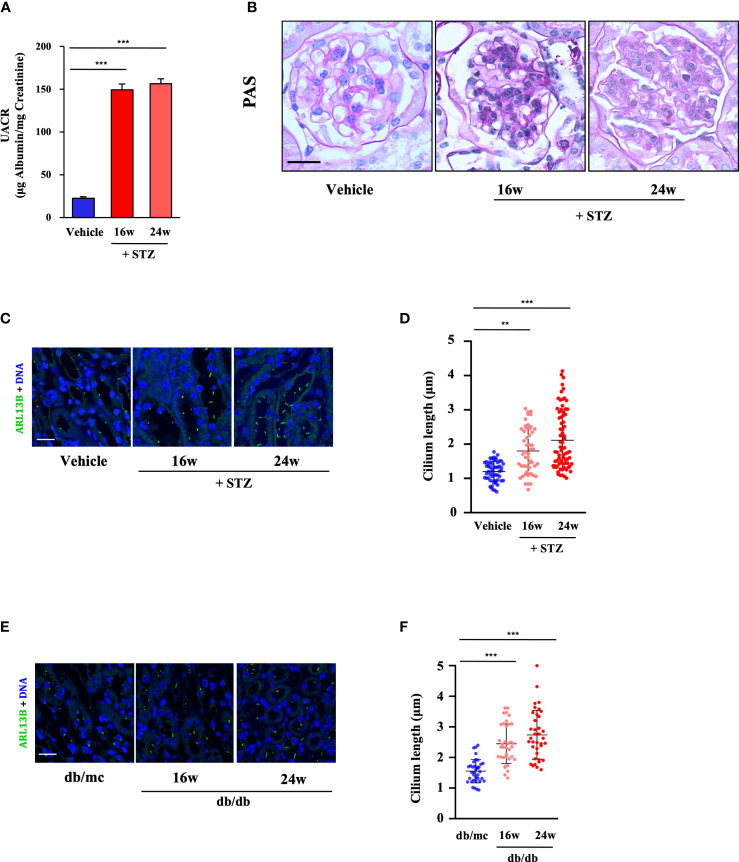
Renal cilia were lengthened in two different diabetic mouse models. **(A)** Urinary albumin-to-creatinine ratio (UACR) at 0, 16, and 24 weeks post-STZ injection (four mice per group). **(B)** Kidney sections from STZ-induced diabetic mice at 0, 16, and 24 weeks post-STZ injection were stained with PAS. Scale bar, 20 μm. **(C)** Kidney sections from STZ-induced diabetic mice were stained for the ciliary marker ARL13B (green) and DNA (blue). Scale bar, 20 μm. **(D)** Quantification of the cilium length in **(C)** (four mice per group). **(E)** Kidney sections from db/db mice were stained for the ciliary marker ARL13B (green) and DNA (blue). Scale bar, 20 μm. **(F)** Quantification of the cilium length in **(E)** (four mice per group). Each dot represents one cell. The data are the means ± SD. One-way ANOVA was performed followed by Dunnett’s multiple comparisons. **P < 0.01; ***P < 0.001.

Images were acquired at room temperature with a 60×/1.42 oil objective (**Figures 1A**, **2C, E**) on an Olympus FV1000 or a 100×/1.42 oil object (**Figure 1E**) on a Nikon Superresolution Microscope (N-SIM). All acquisition settings were kept constant for the experimental and control groups in the same experiment. All raw images were analyzed with Volocity 6.0 software (PerkinElmer). The cilium length was measured from the tip of the cilium to the base.

#### 2.5.1 Kidney histology

Kidney samples were fixed overnight in 4% PFA, embedded in paraffin, and sectioned to a thickness of 4 μm. Sections were stained with periodic acid–Schiff (PAS) to analyze the glomerular area and mesangial matrix expansion. Mesangial expansion was defined as a PAS-positive and nuclei-free area in the mesangium. Images were captured using a dotSlide 2.1 virtual microscopy system (Olympus).

#### 2.5.2 Statistical analysis

The statistically analyzed data are expressed as the mean ± SD. A standard two-tailed unpaired Student’s t-test was used to analyze two groups. To compare the means between three groups, one-way ANOVA followed by Dunnett’s multiple comparison test was used, as noted in the figure legends. Statistical significance was achieved when *P* < 0.05. For all tests, differences were considered statistically significant if *P*-values were < 0.05 (as indicated with *; **P < 0.01; ***P < 0.001). We performed the statistical analysis using GraphPad Prism. Investigators were blinded during the assessment of all staining assays.

## 3 Results

### 3.1 Aberrant ciliogenesis and a dramatic increase in cilium length in humans with DKD

To examine the role of cilia in DKD, we first examined cilia formation in the kidney biopsies of patients at different clinical stages (**Supplementary Table S1**) by staining for the ciliary marker ARL13B (**Figure 1A**). The occurrence of ciliogenesis in RTECs was positively correlated with different DKD classes by PAS analysis (**Figure 1B**). Our data showed that approximately 5% of RTECs in healthy controls formed cilia, whereas 78% of RTECs in DKD patients exhibited abnormal ciliogenesis (**Figure 1C**), and increases in ciliated cells (**Figure 1D**) and the cilium length (**Figures 1E, F**) were positively correlated with the progression of DKD. Compared with 2.42 μm in Class I, the mean cilium length increased to 5.01 μm in Class III. These data suggested that the abnormal ciliogenesis in RTECs might be the cause of continuous glomerular hyperfiltration in the lumen of the renal tubules.

### 3.2 Renal primary cilia lengthen in two different models of diabetic mice

To further validate these results, we examined the cilia occurrence and length in RTECs from diabetic mice at 0, 16, and 24 weeks. Compared with the control group, STZ-induced diabetic mice showed increased levels of urine albumin-to-creatinine ratio (UACR) (**Figure 2A**). Consistent with impaired kidney function, histological changes by PAS analysis, including glomerular sclerosis and mesangial matrix expansion, were more severe in diabetic mice with time post-STZ injection than in control groups (**Figure 2B**).

Subsequently, we analyzed the cilia formation in STZ-induced diabetic mice (at 16 and 24 weeks post-STZ injection), and the results showed that primary cilia in the RTECs in diabetic mice were much longer at 24 weeks post-STZ injection than those in the 16-week and vehicle groups. Compared with 1.12 μm in the control group, the mean cilium length increased to 2.11 μm at 24 weeks post-STZ injection (**Figures 2C, D**). Additionally, similar results were observed in the RTECs of db/db mice; the mean cilium length of db/db mice increased to 2.74 μm at 24 weeks compared with db/mc mice with 1.55 μm (**Figures 2E, F**).

Taken together, our findings identified abnormal ciliogenesis and an increase in the cilium length in the kidneys of DKD patients, and the cilium length was positively correlated with the DKD class. Two different diabetic mouse models also confirmed the correlation between primary cilia and DKD progression.

## 4 Discussion

The data from the kidney biopsies of DKD patients indicate that the cilium length is more than doubled throughout the renal tubule and the collecting duct in Class III compared with Class I. Elongation of the cilium length was also observed in two different diabetic mouse models. It has been shown that the expression of Sirtuin-1 (SIRT1), a NAD^+^-dependent protein/histone deacetylase, is reduced in the human diabetic kidney (21). HDAC6, a tubulin deacetylase, promotes ciliary disassembly by destabilizing the ciliary axoneme (22, 23). The expression and acetylation of HDAC6 are regulated by Sirt1 (24), which downregulates the expression of HDAC6, and the activity of tubulin deacetylation is inhibited, thus promoting the aberrant ciliogenesis. Therefore, we hypothesized that HDAC6-dependent tubulin deacetylation was inhibited in DKD. The possible effect of aberrant ciliogenesis and the increase in the cilium length in RTECs still need to be examined in future studies, and aberrant ciliogenesis may influence mitochondrial biogenesis and fatty acid β oxidation, accelerating renal fibrosis.

Orhon et al. demonstrated that fluid flow induced autophagy in cultured mammalian kidney epithelial cells through the primary cilium (25). The autophagic response to flow depends on the presence of functional cilia and is necessary for cell shrinkage. Since glomerular hyperfiltration contributes to the initiation of DKD, we hypothesized that tubular damage in DKD mainly resulted from glomerular hyperfiltration, which stimulated the aberrant ciliogenesis and elongated the cilium length.

Primary cilia coordinate a variety of signaling pathways, including the Hedgehog, GPCR, Wnt, and TGF-β signaling pathways, which are important not only in kidney injury, repair, and fibrosis but also during embryonic kidney formation and development (26). The activation of sonic Hedgehog (Shh) contributes to kidney fibrosis and promotes fibroblast proliferation (27). The shortening of primary cilia by TGF-β signaling is related to epithelial-to-mesenchymal transition in mouse renal epithelial cells (28). Persistent cilia dysfunction plays a role in the early stages and progression of renal diseases, such as polycystic kidney disease, NPHP, BBS and MKS (29). Acute tubular necrosis causes an increase in the length of renal cilia, possibly modifying sensory sensitivity during repair (30). Additionally, the length of primary cilia was increased in response to renal ischemia/reperfusion injury and oxidative stress and was restored to normal levels during recovery (31). Therefore, we hypothesized that the increase in the cilium number and length in DKD accelerates ciliary sensitivity by augmenting cilium-based signal transduction, which then inhibits the activity of AMPK and dysregulates lipid metabolism and ATP production, leading to significant fibrosis.

Current treatment for DKD is limited to strict glycemic control and blood pressure control with ACE inhibitors or ARBs and SGLT2 inhibitors (32). Our study expands the possible therapeutic targets to include cilia, which is an avenue for the treatment of DKD. Nanoparticles have been used to remotely control cilia movement using an external magnetic field (33). Tissue-specific mRNA delivery and CRISPR-Cas gene editing lipid nanoparticles have been developed (34). Therefore, lipid nanoparticles targeting the renal cilium in DKD are a promising treatment for DKD.

In summary, our work improves the understanding of fundamental cilia biology in kidney disease and is crucial for developing new treatment approaches to alleviate kidney fibrosis and damage.

## Data availability statement

The original contributions presented in the study are included in the article/**Supplementary Material**. Further inquiries can be directed to the corresponding authors.

## Ethics statement

The studies involving human participants were reviewed and approved by Medical Ethics Committee of the First Affiliated Hospital of Xiamen University. The patients/participants provided their written informed consent to participate in this study. The animal study was reviewed and approved by Ethics Committee of Chinese PLA General Hospital. Written informed consent was obtained from the owners for the participation of their animals in this study.

## Author contributions

X-MC, QH, and G-YC supervised the project; Y-FB designed and carried out most of the experiments; PL and C-TW carried out the statistics. BF and S-YC provided reagents and suggestions; J-NL analyzed the data; LZ provided clinical samples; Y-FB, QH and X-FS wrote the paper. All authors discussed the results and commented on the manuscript. All authors contributed to the article and approved the submitted version.

## Funding

This work was supported by the National Natural Science Foundation of China (Grant No. 32141005, 82070741, 82270758, 81900657, 82200797) and Natural Science Foundation of Fujian Province (Grant No. 2020J011243)

## Conflict of interest

The authors declare that the research was conducted in the absence of any commercial or financial relationships that could be construed as a potential conflict of interest.

## Publisher’s note

All claims expressed in this article are solely those of the authors and do not necessarily represent those of their affiliated organizations, or those of the publisher, the editors and the reviewers. Any product that may be evaluated in this article, or claim that may be made by its manufacturer, is not guaranteed or endorsed by the publisher.
