# Supplementary material for: Renal primary cilia lengthen in the progression of diabetic kidney disease
Source: Front Endocrinol (Lausanne). 2022 Nov 17;13:984452. doi: 10.3389/fendo.2022.984452 (PMC9713695; doi:10.3389/fendo.2022.984452)
Supplement: Supplementary file 1 [file Presentation_1.pptx]

## Slide 1
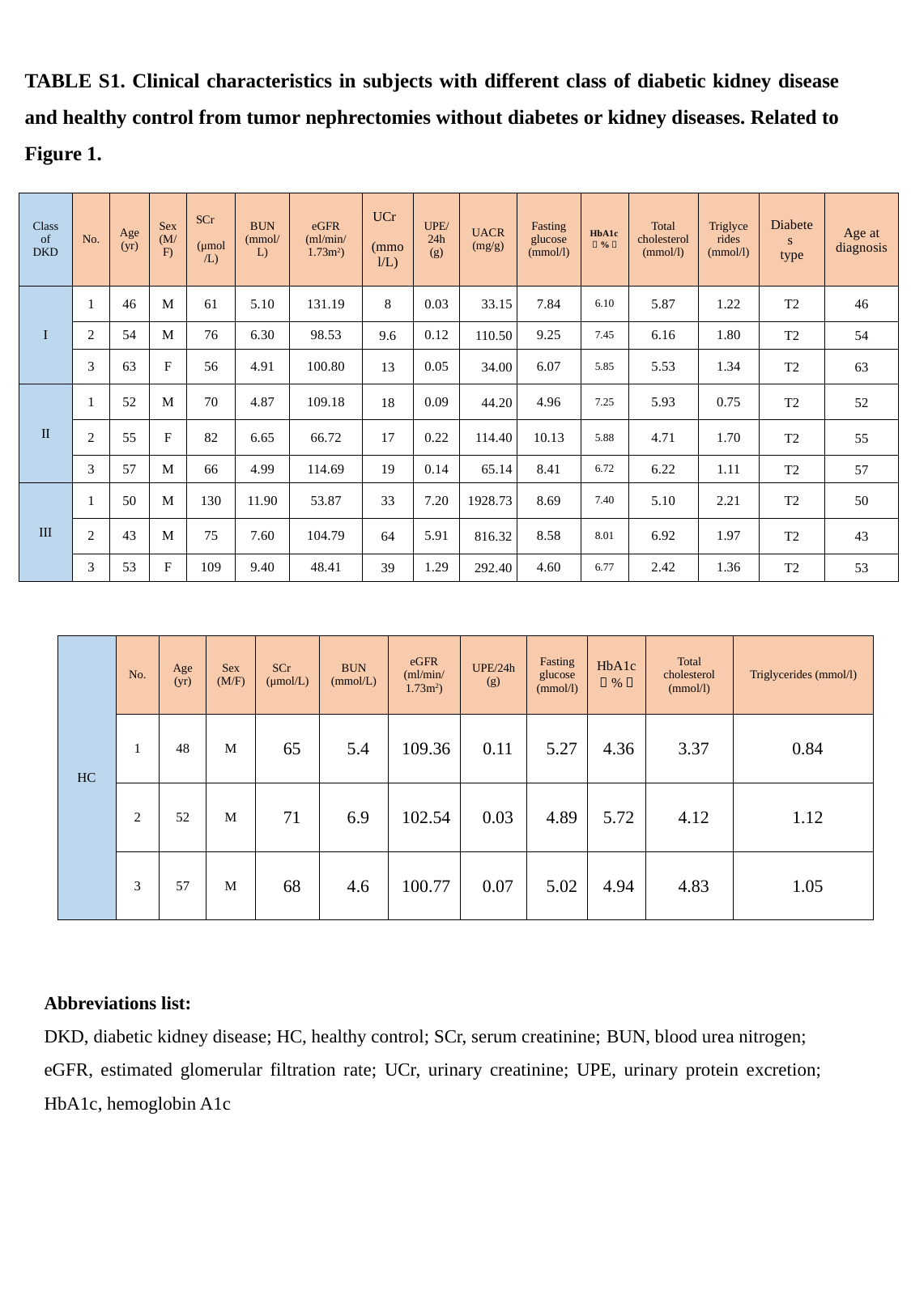

TABLE S1. Clinical characteristics in subjects with different class of diabetic kidney disease and healthy control from tumor nephrectomies without diabetes or kidney diseases. Related to Figure 1.
| Class of DKD | No. | Age (yr) | Sex (M/F) | SCr (μmol/L) | BUN (mmol/L) | eGFR (ml/min/ 1.73m2) | UCr (mmol/L) | UPE/24h (g) | UACR (mg/g) | Fasting glucose (mmol/l) | HbA1c（%） | Total cholesterol (mmol/l) | Triglycerides (mmol/l) | Diabetes type | Age at diagnosis |
| --- | --- | --- | --- | --- | --- | --- | --- | --- | --- | --- | --- | --- | --- | --- | --- |
| I | 1 | 46 | M | 61 | 5.10 | 131.19 | 8 | 0.03 | 33.15 | 7.84 | 6.10 | 5.87 | 1.22 | T2 | 46 |
| | 2 | 54 | M | 76 | 6.30 | 98.53 | 9.6 | 0.12 | 110.50 | 9.25 | 7.45 | 6.16 | 1.80 | T2 | 54 |
| | 3 | 63 | F | 56 | 4.91 | 100.80 | 13 | 0.05 | 34.00 | 6.07 | 5.85 | 5.53 | 1.34 | T2 | 63 |
| II | 1 | 52 | M | 70 | 4.87 | 109.18 | 18 | 0.09 | 44.20 | 4.96 | 7.25 | 5.93 | 0.75 | T2 | 52 |
| | 2 | 55 | F | 82 | 6.65 | 66.72 | 17 | 0.22 | 114.40 | 10.13 | 5.88 | 4.71 | 1.70 | T2 | 55 |
| | 3 | 57 | M | 66 | 4.99 | 114.69 | 19 | 0.14 | 65.14 | 8.41 | 6.72 | 6.22 | 1.11 | T2 | 57 |
| III | 1 | 50 | M | 130 | 11.90 | 53.87 | 33 | 7.20 | 1928.73 | 8.69 | 7.40 | 5.10 | 2.21 | T2 | 50 |
| | 2 | 43 | M | 75 | 7.60 | 104.79 | 64 | 5.91 | 816.32 | 8.58 | 8.01 | 6.92 | 1.97 | T2 | 43 |
| | 3 | 53 | F | 109 | 9.40 | 48.41 | 39 | 1.29 | 292.40 | 4.60 | 6.77 | 2.42 | 1.36 | T2 | 53 |
| HC | No. | Age (yr) | Sex (M/F) | SCr (μmol/L) | BUN (mmol/L) | eGFR (ml/min/ 1.73m2) | UPE/24h (g) | Fasting glucose (mmol/l) | HbA1c（%） | Total cholesterol (mmol/l) | Triglycerides (mmol/l) |
| --- | --- | --- | --- | --- | --- | --- | --- | --- | --- | --- | --- |
| | 1 | 48 | M | 65 | 5.4 | 109.36 | 0.11 | 5.27 | 4.36 | 3.37 | 0.84 |
| | 2 | 52 | M | 71 | 6.9 | 102.54 | 0.03 | 4.89 | 5.72 | 4.12 | 1.12 |
| | 3 | 57 | M | 68 | 4.6 | 100.77 | 0.07 | 5.02 | 4.94 | 4.83 | 1.05 |
Abbreviations list:
DKD, diabetic kidney disease; HC, healthy control; SCr, serum creatinine; BUN, blood urea nitrogen;
eGFR, estimated glomerular filtration rate; UCr, urinary creatinine; UPE, urinary protein excretion; HbA1c, hemoglobin A1c

## Slide 2
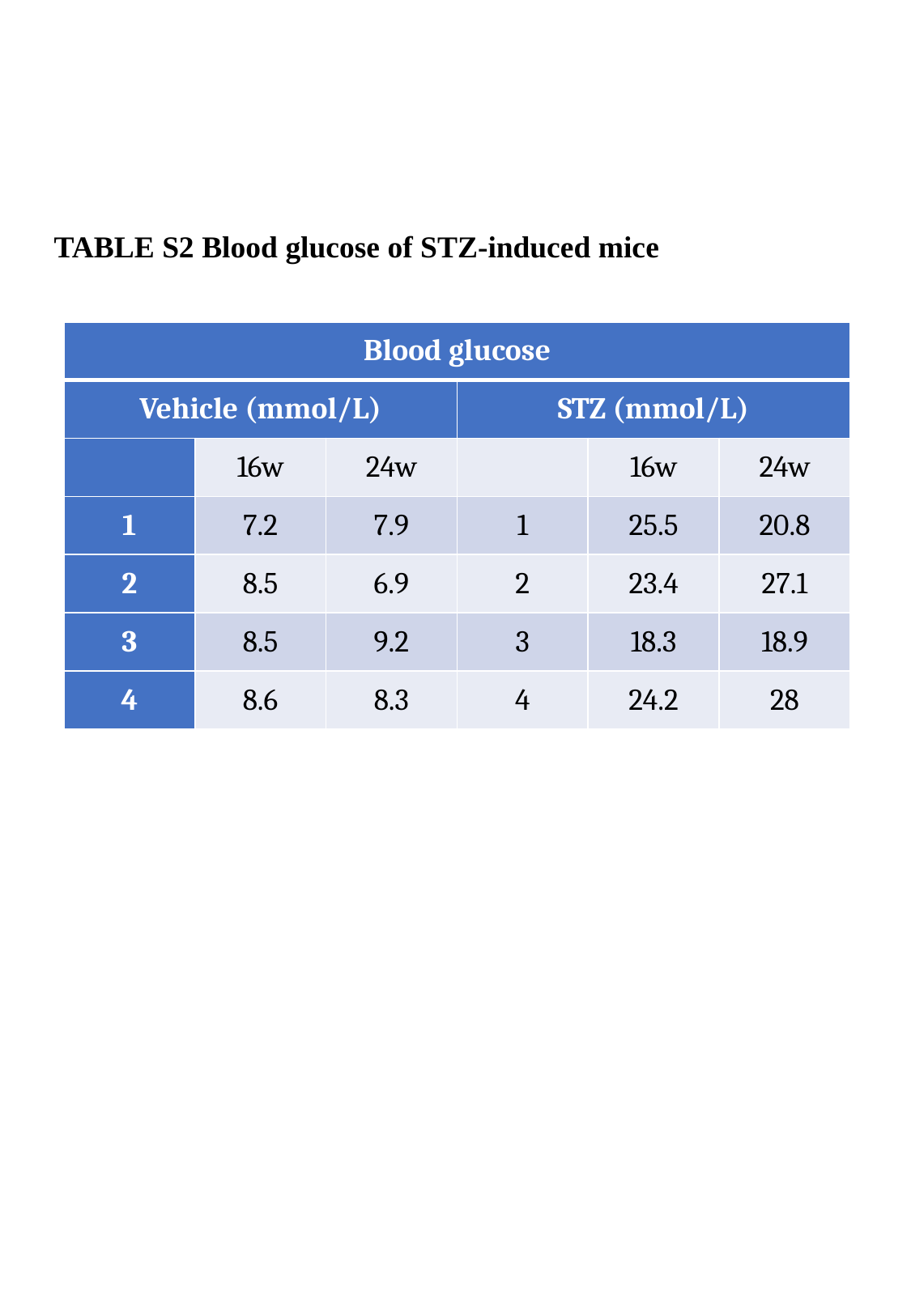

TABLE S2 Blood glucose of STZ-induced mice
| Blood glucose | | | | | |
| --- | --- | --- | --- | --- | --- |
| Vehicle (mmol/L) | | | STZ (mmol/L) | | |
| | 16w | 24w | | 16w | 24w |
| 1 | 7.2 | 7.9 | 1 | 25.5 | 20.8 |
| 2 | 8.5 | 6.9 | 2 | 23.4 | 27.1 |
| 3 | 8.5 | 9.2 | 3 | 18.3 | 18.9 |
| 4 | 8.6 | 8.3 | 4 | 24.2 | 28 |

## Slide 3
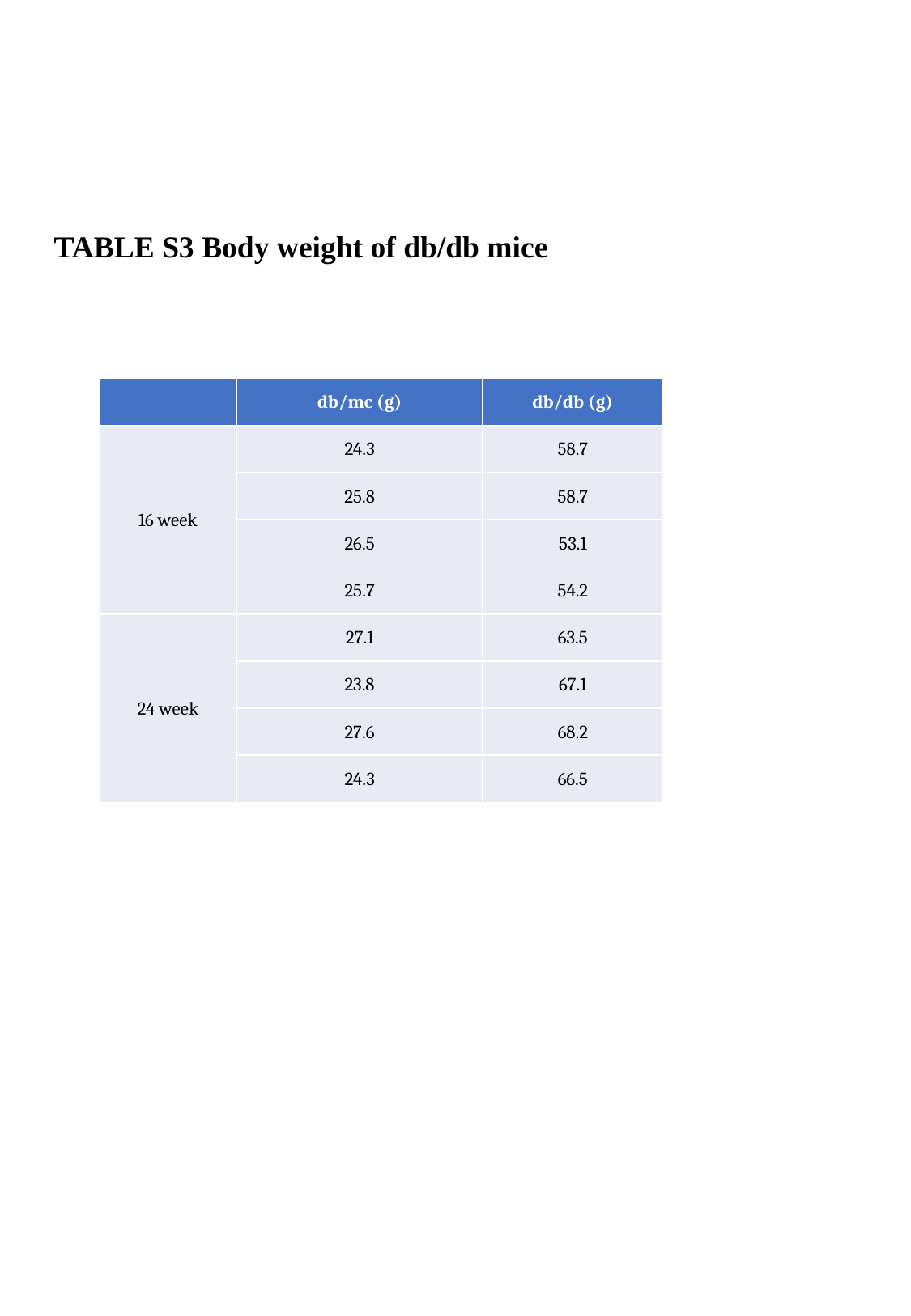

TABLE S3 Body weight of db/db mice
| | db/mc (g) | db/db (g) |
| --- | --- | --- |
| 16 week | 24.3 | 58.7 |
| | 25.8 | 58.7 |
| | 26.5 | 53.1 |
| | 25.7 | 54.2 |
| 24 week | 27.1 | 63.5 |
| | 23.8 | 67.1 |
| | 27.6 | 68.2 |
| | 24.3 | 66.5 |
